# *Daphnia *Halloween genes that encode cytochrome P450s mediating the synthesis of the arthropod molting hormone: Evolutionary implications

**DOI:** 10.1186/1471-2148-8-60

**Published:** 2008-02-25

**Authors:** Kim F Rewitz, Lawrence I Gilbert

**Affiliations:** 1 Department of Science, Systems and Models, Roskilde University, Postbox 260, DK-4000 Roskilde, Denmark; 2 Department of Biology, University of North Carolina, Chapel Hill, NC 27599-3280 USA

## Abstract

**Background:**

In crustaceans and insects, development and reproduction are controlled by the steroid hormone, 20-hydroxyecdysone (20E). Like other steroids, 20E, is synthesized from cholesterol through reactions involving cytochrome P450s (CYPs). In insects, the CYP enzymes mediating 20E biosynthesis have been identified, but evidence of their probable presence in crustaceans is indirect, relying solely on the ability of crustaceans to synthesize 20E.

**Results:**

To investigate the presence of these genes in crustaceans, the genome of *Daphnia pulex *was examined for orthologs of these genes, the Halloween genes, encoding those biosynthetic CYP enzymes. Single homologs of *spook-CYP307A1*, *phantom-CYP306A1*, *disembodied-CYP302A1*, *shadow-CYP315A1 *and *shade-CYP314A1 *were identified in the *Daphnia *data base. Phylogenetic analysis indicates an orthologous relationship between the insect and *Daphnia *genes. Conserved intron/exon structures and microsynteny further support the conclusion that these steroidogenic CYPs have been conserved in insects and crustaceans through some 400 million years of evolution.

**Conclusion:**

Although these arthropod steroidogenic CYPs are related to steroidogenic CYPs in *Caenorhabditis elegans *and vertebrates, the data suggest that the arthropod steroidogenic CYPs became functionally specialized in a common ancestor of arthropods and are unique to these animals.

## Background

Steroid hormones, regulate essential processes during development and reproduction, and are synthesized from cholesterol under the control of steroidogenic enzymes in the cytochrome P450 (CYP) family [1]. In *Caenorhabditis elegans*, insects and vertebrates, different steroids are produced to control developmental processes, suggesting that steroidogenic CYPs evolved and became functionally specialized in different lineages during evolution. In insects, a specific biosynthetic pathway yielding 20-hydroxyecdysone (20E), the arthropod molting hormone, evolved, whereas in the line leading to vertebrates, biosynthetic CYPs that produce the vertebrate-type steroids evolved [2]. Since there is some evidence of the presence of vertebrate-type sex steroids in invertebrates such as echinoderms and mollusks, although no unequivocal evidence that they can synthesize these steroids [3], the possibility remains that CYPs with the capacity to produce vertebrate-type sex steroids were present in the common ancestor even before the protostome-deuterostome split. Thus, the evolution of steroidogenic CYPs is still an open question.

**Figure 1:**
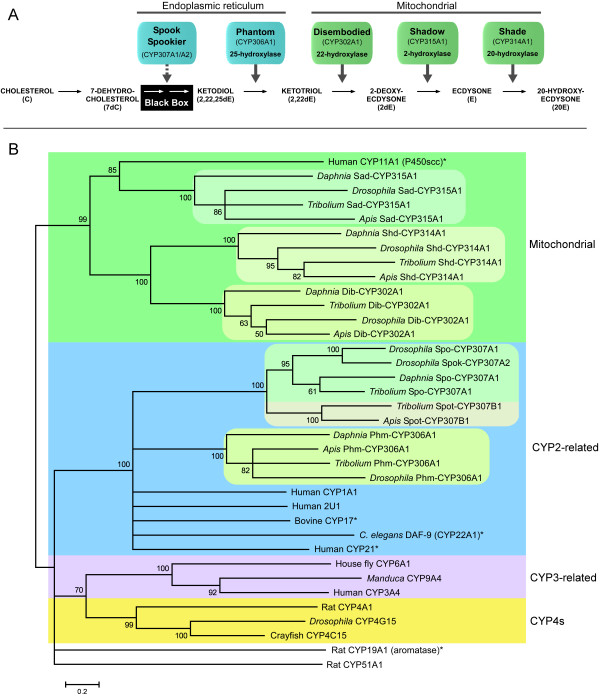
**Scheme of 20-hydroxyecdysone (20E) biosynthesis and a phylogenetic tree including *Daphnia *Halloween orthologs**. A) Biosynthetic scheme showing the steroidogenic CYP enzymes encoded by genes in the Halloween family mediating steps in the conversion of cholesterol to 20E and the subcellular distribution of these enzymes. The Black Box denotes an uncharacterized series of oxidative modifications converting 7-dehydrocholesterol (7dC) to the first ecdysteroid-like molecule, namely the ketodiol (2,22,25-trideoxyecdysone: 2,22,25-dE) [1]. Dashed arrow indicates that there is no direct evidence for the catalytic function of Spook (Spo) and Spookier (Spok), but several pieces of evidence point to a function of these enzymes in the Black Box [16]. 2,22-dideoxyecdysone (2,22-dE). B) Maximum Likelihood phylogenetic tree showing relationships of the *Daphnia *sequences with orthologs of the insect steroidogenic CYP products of the Halloween genes Spo, Spok, Spookiest (Spot), Phantom (Phm), Disembodied (Dib), Shadow (Sad), Shade (Shd). Other selected vertebrate and *C. elegans *steroidogenic and non-steroidogenic CYPs are included to infer relationship to major classes of CYPs. Members of major metazoan CYP classes are represented in this analysis: the mitochondrial, CYP2-related, CYP3-related, CYP4s, CYP19 and CYP51. Numbers indicate support values obtained by bootstrapping 100 replicates and branches under the threshold value of 50 are shown as polytomies. Human CYP3A4 (AAI01632), Rat CYP4A1 (NP_787031), Crayfish CYP4C15 (AAF09264), *Drosophila *CYP4G15 (AAF76522) House fly CYP6A1 (AAA29293), *Manduca *CYP9A4 (AAD51036), Human CYP11A1 – P450scc cholesterol side chain cleavage enzyme (AAH32329), Bovine CYP17A1 – steroid 17α-hydroxylase/17,20 lyase (P05185), Rat CYP19A1 – aromatase converting androgens to estrogens (P22443), Human CYP21 – steroid 21-hydroxylase (AAB59440), Human CYP1A1 (AAH23019), Human CYP2U1 (NP_898898), Rat CYP51 – sterol 14α-demethylase (Q64654), and *C. elegans *DAF-9 (CYP22A1) – produces the steroid ligand for the DAF-12 nuclear receptor (AAL65132) [35]. * Shows CYP enzymes from animals, other than arthropods, that are involved in steroidogenesis.

Crustaceans are believed to represent the ancestral arthropods from which insects originated [4]. The evolutionary relationship between these two groups is evident from the common growth strategy of insects and crustaceans that involves molting so that growth can occur. Molting is governed by periodic increases in the levels of 20E that elicit the programs that coordinate the developmental and metamorphic transitions [5]. Although a great deal of evidence reveals that crustaceans, like insects, synthesize 20E from cholesterol [6], the molecular details of steroidogenesis in crustaceans remain conjectural. In insects, steroidogenic CYPs are products of the Halloween genes *phantom *(*phm*: *CYP306A1*), *disembodied *(*dib*: *CYP302A1*), *shadow *(*sad*: *CYP315A1*) and *shade *(*shd*: *CYP314A1*) and are responsible for the last four hydroxylations in the pathway leading to 20E [7-14] that is biochemically similar to one that yields 20E in crustaceans [6] (Fig. 1). In *Drosophila melanogaster*, mutations in these genes disrupt 20E production and cause the arrest of embryonic development and death. *spook *(*spo*: *Cyp307a1*) is another member of this CYP group which when mutated results in low 20E mutants [15,16] and is believed to mediate a yet uncharacterized step (the Black Box) in the biosynthesis of 20E preceding those of Phm, Dib, Sad and Shd. In contrast to *phm*, *dib*, *sad *and *shd *for which each insect genome carries one ortholog, several paralogs of *spo*-like (*CYP307*) genes have been formed by duplications, which in turn have evolved lineage-specific complements of these genes [2,16,17]. For example, *Drosophila *has two *spo*-like genes, *spo *and *spookier *(*spok*: *Cyp307a2*) [16]. These two genes are close paralogs that are believed to mediate the same enzymatic reaction, although at different stages of development.

Although one would expect that orthologs of the insect Halloween genes are present in crustaceans, there is no molecular evidence for the existence of these genes in crustaceans. We have tried for several years to probe hexapod crustaceans for Halloween gene orthologs under various hormonal regimens using degenerate primers based on the *Drosophila *and *Bombyx *genes, but we have been unsuccessful (K.F. Rewitz, J.T. Warren, E. Chang and L.I. Gilbert). The development of the genome data base of the more primitive crustacean, *Daphnia pulex*, allowed us to survey this genome and conduct phylogenetic analyses that suggest strongly that orthologs of *spo*, *phm*, *dib*, *sad *and *shd *do exist in *Daphnia *and thus, in a crustacean i.e. the genes appeared in arthropods before the radiation of insects.

## Results and Discussion

By searching the *Daphnia *data bases [18,19] we obtained candidate sequences for orthologs of the insect Halloween genes in *Daphnia*. Single orthologs of *phm*, *dib*, *sad *and *shd *were retrieved and only one sequence exhibited significant similarity to the *spo*-like genes in the CYP307 family. We also searched the genomes of non-arthropod invertebrates including the cnidarian *Nematostella vectensis*, the nematodes *C. elegans *and *Brugia malayi*, the annelid *Capitella capitata*, the mollusk *Lottia gigantea*, the echinoderm *Strongylocentrotus purpuratus *[19,20] for Halloween gene orthologs using TBLASTN. Analyses of these invertebrate genomes did not result in any significant hits indicating the absence of Halloween orthologs and neither have we been able to identify orthologs of these genes in any of several vertebrate species.

The genes obtained from *Daphnia *encode approximately 500 amino acid open reading frames (ORFs) which is typically for proteins belonging to the CYP family [21]. Alignment of *Daphnia *sequences with orthologs of the insect Halloween genes shows that the genes, in addition to being conserved in areas that comprise canonical structural CYP motifs, exhibit considerable conservation in regions that are believed to determine substrate specificity (Additional file 1). This indicates that the genes are functionally conserved in *Daphnia *as they are in insects [2]. The overall amino acid identity between deduced orthologous proteins from eight insects belonging to four different orders and the *Daphnia *orthologs ranges from an average of 55.2% ± 6.8 SD (standard deviation) for Spo-like proteins to somewhat lower values for Sad proteins (38.7% ± 8.8 SD). Thus, *spo*-like genes are the most highly conserved of these genes and this appears to be true for *Daphnia *like it is for insects. Conservation of these genes from insect to crustaceans, which separated ~400 millions years ago [4], shows that selection has preserved the genes because of their function. The reason that *spo*-like genes are more conserved than the other arthropod steroidogenic CYP enzymes is not known, although it may be related to the possibility that Spo acts in the rate-limiting Black Box reaction(s) [5,22]. If Spo is involved in control of the flux through the pathway it may have been a particular target for selection because mutations altering its enzyme activity would have had increased consequences compared to mutations altering the activity of enzymes that are less rate-limiting. A phylogenetic analysis was performed using the *Daphnia *sequences.

### Phylogenetic analysis of Daphnia candidate orthologs

A phylogenetic tree was constructed with *Daphnia *sequences retrieved from the BLAST searches and orthologs of the insect Halloween genes from *Drosophila*, red flour beetle *Tribolium castaneum *and honey bee *Apis mellifera*, which represent three different orders (Diptera, Coleoptera and Hymenoptera, respectively) of holometabolous insects. The *Daphnia *gene products separate with the insect orthologs of Spo/Spok, Phm, Dib, Sad and Shd in this phylogenetic tree with high bootstrap support (Fig. 1). In examining the genome of *Daphnia *for orthologs of these genes, we noticed no closely related paralogs to the obtained candidate orthologs. This supports the orthology of the genes and indicates that the genes became functionally specialized before the split between crustaceans and insects and have been under heavy selection pressure ever since. Only one *spo*-like gene was obtained and this gene is phylogenetically a *CYP307A *subfamily gene i.e. most closely related to *spo*/*spok*. No ortholog of *spookiest *(*spot*: *CYP307B1*) was found indicating that this gene does not exist in *Daphnia*, which implies that it is insect-specific i.e. originating from a duplication occurring after insects arose from crustaceans [4] or that it was lost in *Daphnia *as it has been in some insects e.g. lepidopterans and *Drosophila *species [2].

In the phylogenetic analysis, sequences of steroidogenic CYPs from vertebrates and *C. elegans *were included to probe ancestral relationships and the origin of CYPs involved in steroid biogenesis (Fig. 1). Figure 1 includes major groups of metazoans CYPs i.e. mitochondrial, CYP2-related, CYP3-related, CYP4-related, CYP19s and CYP51s. The steroidogenic CYPs of insects and their *Daphnia *orthologs are evolutionarily related to vertebrate and *C. elegans *steroidogenic CYPs since they cluster in two major groups, those related to CYP2 enzymes (Spo, Phm, CYP17, CYP21 and DAF-9) and those that are mitochondrial (Dib, Sad, Shd and CYP11A1). Therefore, it is likely that different steroidogenic CYP enzymes are derived from common ancestors and were recruited for steroid biosynthesis prior to the protostome-deuterostome split. Evolution of these ancestral CYPs in *C. elegans*, arthropods and vertebrates, by gene duplication and divergence, likely shaped the biosynthetic pathways yielding the different groups of steroids in these animals.

### Conservation of gene structure

**Figure 2:**
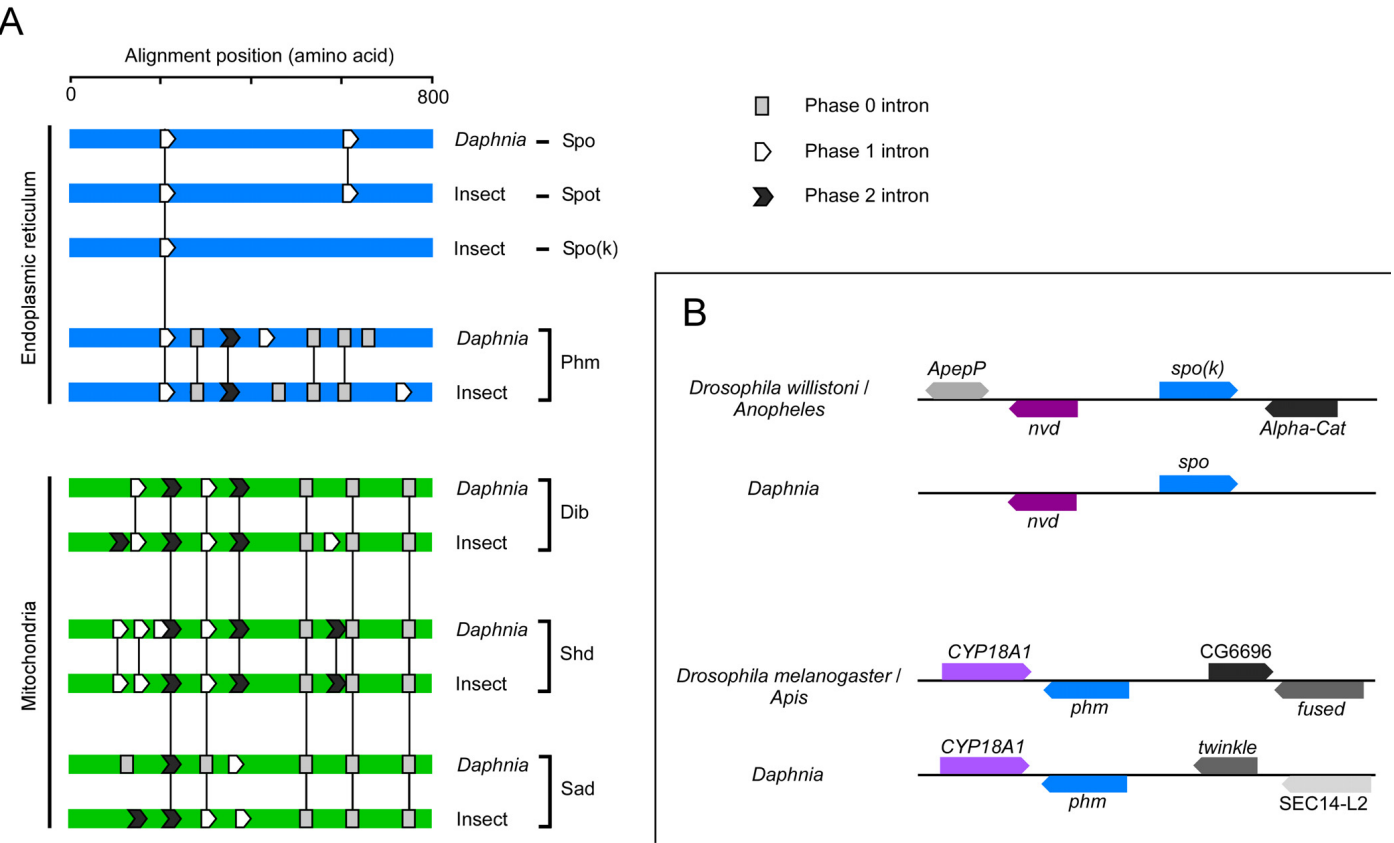
**Intron/exon structure and microsynteny of *Daphnia *and insect Halloween genes**. A) Introns are mapped on the aligned protein sequences of Spo (CYP307A1), Spot (CYP307B1), Spok (CYP307A2), Phm (CYP306A1), Dib (CYP302A1), Sad (CYP315A1), Shd (CYP314A1). Introns located at the same position and in the phase (the nucleotide position of the intron within a codon: phase 0 between codons, phase 1 after the first base and phase 2 after the second base), on the aligned proteins, are shown as conserved by connecting vertical lines. Insect introns represent introns found in species of insects previously described [2], except *Tribolium shd *which exhibits unique introns that are not shown. B) Preserved microsynteny in local genome regions surrounding *phm *and *spo(k)*. The arrangement of *phm *and its paralog *CYP18A1 *is conserved in insects and *Daphnia *and so is the microsyntenic relationship of *spo *(*spok *in *Drosophila*) and *neverland *(*nvd*). Nvd is believed to be involved in the conversion of cholesterol to 7-dehydrocholesterol (7dC), the step preceding the Black Box in which Spo/Spok may function [16,26]. The transcriptional direction of the genes is shown by arrowheads. The double arrowhead indicates that *ApepP *is in opposite orientations in *Drosophila *and *Anopheles*.

**Figure 3:**
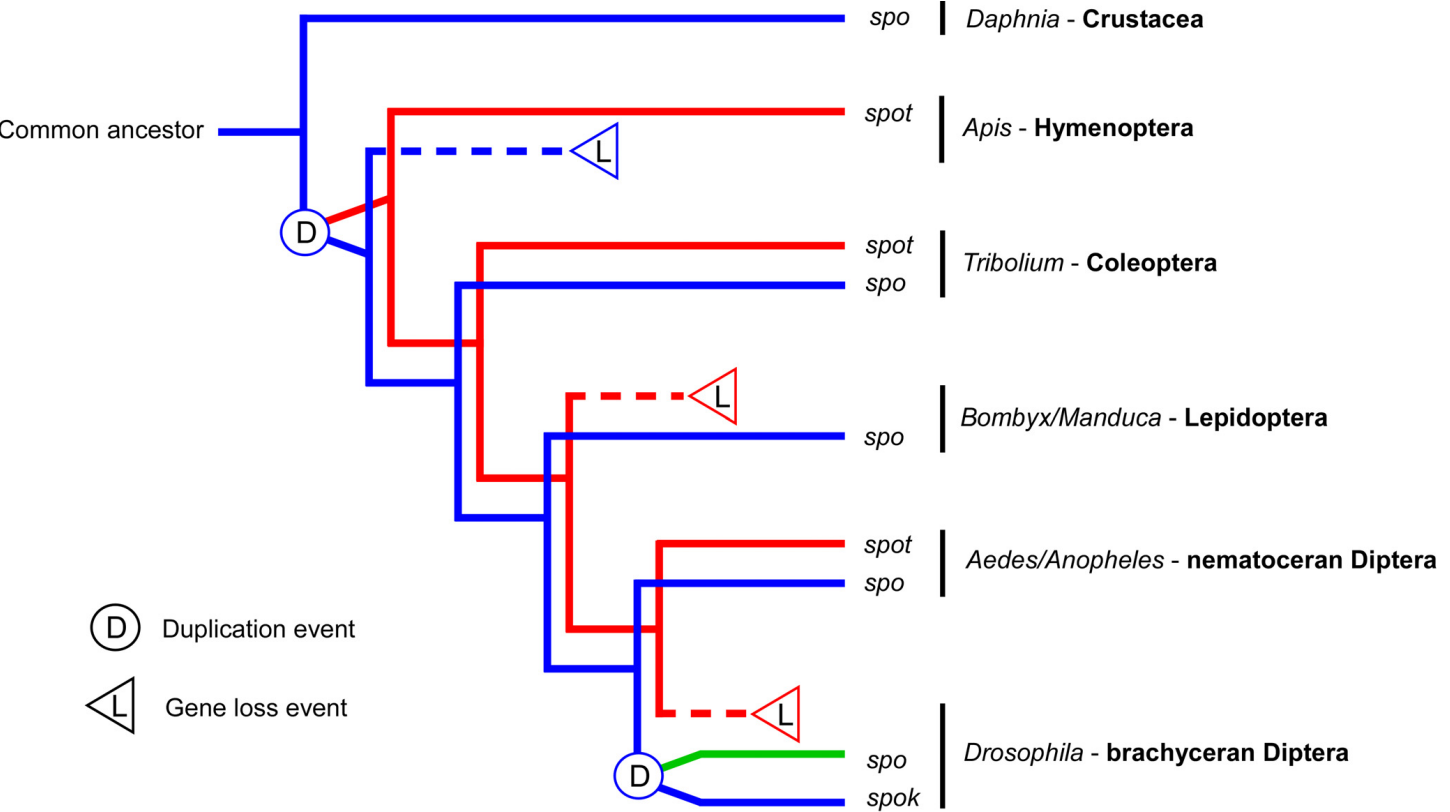
**Lineage-specific duplications and losses of *spo*-like genes in *Daphnia *and insects**. An evolutionary scenario based on the observed distribution of *spo*-like (*CYP307*) genes in arthropods. Since insects are believed to have evolved from crustaceans living in freshwater environments [4] and the only *spo*-like gene observed in *Daphnia *belongs to the *CYP307A *subfamily, the ancestral arthropod *spo *gene was likely a *CYP307A *gene. An early duplication, which probably occurred after insects diverged from crustaceans, gave rise to *spo *(*CYP307A1) *and *spot (CYP307B1)*. In *Drosophila*, the ancestral *spo*-like gene, referred to as *spok (Cyp307a2)*, underwent a second round of duplication in which an intronless retrogene arose. In *Drosophila *this gene is referred to as *spo*. Note that another round of gene duplication, which occurred in the line of *Drosophila *evolution leading to the subgenus Drosophila [17], is not shown.

Comparison of intron positions of the aligned *Daphnia *protein sequences and the insect orthologs of the Halloween genes shows that many introns are conserved (Fig. 2). Each *Daphnia *ortholog contains conserved intron/exon organization typical of their respective insect orthologs. The conserved gene structure suggests that the *Daphnia *genes have a common evolutionary origin with the insect Halloween genes and support the inferred phylogenetic relationship suggesting orthology (Fig. 1). In *phm*, five introns are conserved among *Daphnia *and insects and only two introns are unique to the *Daphnia *gene. Introns that have no counterparts can be explained by intron gain or alternatively these introns have been lost. Interestingly, *Daphnia spo *has two introns whereas insect *spo *genes have only one. The position and phase (the nucleotide position in a codon) of introns in the *Daphnia spo *gene are equivalent to those conserved in insect *spot *genes. However, the *Daphnia *sequence is the closest ortholog of the insect *spo/spok *genes phylogenetically, and only shares one of these conserved introns. A likely evolutionary scenario is that the ancestral gene was a *spo *gene containing both introns (Fig. 3). In the insect lineage, after the split from crustaceans, duplication of this gene resulted in two copies that diverged into *spo *and *spot *paralogs after which one intron was lost in the insect *spo *genes.

Although several introns are conserved among the mitochondrial *dib*, *sad *and *shd*, introns that are unique to each group of orthologs (e.g. the first phase 1 intron in *dib *and *shd*) indicate an orthologous relationship to the respective groups. The conserved gene structure of these genes suggests that they evolved from a common ancestor of mitochondrial CYPs by duplications and diverged into the steroidogenic enzymes that are functionally specialized to carry out the last three steps in which C22, C2 and C20 hydroxylations form 20E. This implies, at least in part, that the biosynthetic pathway evolved step-wise by duplication and functional divergence of the biosynthetic enzymes. In vertebrates, functional divergence and specialization of steroidogenic CYPs are known from studies of the CYP11 paralogs. CYP11A1 is the enzyme that mediates the removal of the side chain of cholesterol [23]. CYP11B1 and CYP11B2 are paralogs, from a recent duplication, that became functionally specialized as the steroid 11β-hydroxylase and the aldosterone synthase, respectively [24]. *dib*, *sad *and *shd *also exhibit conservation of gene structure when compared to vertebrate mitochondrial CYPs involved in steroidogenesis [2].

### Microsynteny supports orthology

The conserved arrangement of genes on the chromosome of different species (microsynteny) can be used to infer phylogenetic relationships that support the orthology of genes [2,17]. Analysis of local genome structures surrounding the Halloween genes shows little conservation of microsynteny between insects and crustaceans. This is likely due to the relatively great evolutionary time separating insects from crustaceans. However, remnants showing microsynteny can be found in at least two cases. *CYP18A1 *is a paralog of *phm *which is found in *Drosophila *and most other insects [25]. In *Drosophila *and *Apis*, *phm *and *CYP18A1 *are arranged tail-to-tail and adjacent to these genes are CG6696 and *fused *(Fig. 2). In *Daphnia*, the microsyntenic relationship of *phm *and *CYP18A1 *is conserved, although CG6696 and *fused *appear to have been rearranged to different chromosomal locations.

Comparison of gene organization in a region of approximately 100 kb surrounding the *spo(k) *locus reveals that *spo *is adjacent to *neverland *(*nvd*) [26] in *Daphnia *and the mosquito *Anopheles gambiae*. Analysis of the genome region of *spo*-like genes in species of *Drosophila *(available at FlyBase [27]) shows that in *Drosophila pseudoobscura*, *Drosophila willistoni *and *Drosophila mojavensis nvd *is located within 60 kb of *spok*. This is in agreement with the view that *spok *is the ancestral gene in Drosophilidae. Intriguingly, Nvd is a conserved Rieske-like oxygenase believed to be involved in the conversion of cholesterol to 7-dehydrocholesterol in insects and *C. elegans *[26,28]. In insects, this is presumably the first step in the biosynthetic pathway of 20E from cholesterol via 7-dehydrocholesterol, the latter being the substrate that enters the Black Box, in which Spo/Spok likely participate (although at different developmental stages) to produce the substrate for Phm, the ketodiol (Fig. 1). Although unequivocal evidence for the exact function of Nvd and Spo/Spok is lacking, experiments utilizing ecdysteroid precursors to rescue mutant phenotypes (RNAi-induced for *nvd *and *spok*) suggest that in *Drosophila nvd *and *spo*/*spok *genes function in these two succeeding steps in the biochemical pathway [16,26]. However, it must be emphasized that the Black Box may hold more than one reaction and more than one enzyme may be participating in this conversion [5].

## Conclusion

Currently, it is clear that CYPs are involved in steroid biosynthesis in vertebrates and invertebrates. In *C. elegans*, insects and vertebrates, the majority of steroidogenic CYPs are related to one of two groups, the CYP2 enzymes and the mitochondrial CYPs. It is therefore likely that these steroidogenic CYPs evolved from common ancestors which had the capacity to modify cholesterol before the split of these animals. This implies that the distinct biosynthetic pathways in these metazoans, which convert cholesterol to different types of steroids, likely result from lineage-specific evolution of ancestral steroidogenic CYPs. The results suggest strongly that steroidogenic CYP enzymes mediating the biosynthesis of 20E are present in crustaceans and are relegated to the Arthropoda. Therefore, these enzymes probably became functionally specialized in the line leading to arthropods. Although functional characterization of the gene products, such as that done for the insect Halloween genes [1,29], is required to obtain unequivocal evidence of their role in 20E production in *Daphnia*, the present data provide the first evidence for steroidogenic CYP genes in crustaceans as well as the groundwork for future functional genomic analyses in the field of crustacean endocrinology.

## Methods

### Data base search

The *Daphnia pulex *genome sequence (v1.0) made available by the *Daphnia *Genomics Consortium and the DOE Joint Genome Institute [19] was mined for orthologs of the insect Halloween genes using TBLASTN searches. *Apis *and *Tribolium *sequences, previously described in Rewitz et al. [2], were used as queries to identify homologous sequences in the *Daphnia *data base. Sequences of insect Halloween genes, previously described in Rewitz et al. [2], were gleaned from FlyBase [27] and Rene Feyereisen's website for insect CYPs [30]. *C. elegans *and vertebrate CYP protein sequences were acquired from NCBI [20].

### Phylogenetic tree construction, intron positions and microsynteny analysis

Predicted *Daphnia *orthologs of the insect Halloween genes were analyzed using a phylogenetic tree with insect Halloween genes from insects belonging to three different orders, *Apis *(Hymenoptera), *Tribolium *(Coleoptera) and *Drosophila *(Diptera). To infer phylogenetic relationships of these steroidogenic CYPs with other members of this multigene family, steroidogenic and non-steroidogenic CYPs representing some of the major groups of metazoan CYPs were included in this analysis i.e. mitochondrial, CYP2-related, CYP3-related and CYP4s, CYP19 and CYP51. Deduced *Daphnia *protein sequences used in the alignment can be found in Additional file 2. A multiple alignment of the protein sequences was constructed with ClustalX (1.83) [31] and manually edited using SeaView [32]. A phylogenetic tree was constructed from this alignment using the Maximum Likelihood method under the Jones-Taylor-Thornton (JTT) substitution model using PHYML (v2.4.5) [33]. Support values were obtained by bootstrapping 100 replicates. Branches with bootstrap support below 50 were collapsed to form polytomies.

Gene structure, that is, intron position and phase (the nucleotide position of the intron within a codon: phase 0 between codons, phase 1 after the first base and phase 2 after the second base) were predicted from manual annotation of the *Daphnia *genes with support from expressed sequence tags (ESTs) data and by homology to insect Halloween genes for which gene structures are known. Intron position and phase were mapped onto a multiple alignment to investigate conservation of intron/exon structure in relation to phylogeny.

Additional support for orthology of *Daphnia *and insect genes was sought by analyses of microsynteny (conserved order of genes). Genome regions surrounding the candidate Halloween orthologs of *Daphnia *and several insects were inspected for ORFs encoding conserved genes. Putative gene orthologs exhibiting conserved arrangement in relation to the Halloween orthologs were tested for orthology by reciprocal best hit Blast searches in which orthologs of each species were BLAST searched against the genome of the other species to look for best hits (putative orthologs).

## Abbreviations

20E (20-hydroxyecdysone); Dib (Disembodied-CYP302A1); Nvd (Neverland); ORF (open reading frame); Phm (Phantom-CYP306A1); Sad (Shadow-CYP315A1); Shd (Shade-CYP314A1); Spo (Spook-CYP307A1); Spok (Spookier-CYP307A2); Spot (Spookiest-CYP307B1); Spo-like (CYP307 family)

## Authors' contributions

KFR conducted the actual phylogenetic and other analyses in this paper and wrote the first draft. LIG came up with the idea of identifying the Halloween genes in a crustacean, gained access to the *Daphnia *data base, recommended that KFR do the search analyses and helped write the final draft of the manuscript. All authors read and approved the final manuscript.

## Supplementary Material

Additional File 1 Putative substrate recognition sites (SRSs). Click here for file

Additional File 2 Deduced protein sequences of *Daphnia pulex *Spo-CYP307A1, Phm-CYP306A1, Dib-CYP302A1, Sad-CYP315A1 and Shd-CYP314A1. Click here for file
