# Supplementary material for: Daphnia Halloween genes that encode cytochrome P450s mediating the synthesis of the arthropod molting hormone: Evolutionary implications
Source: BMC Evol Biol. 2008 Feb 25;8:60. doi: 10.1186/1471-2148-8-60 (PMC2276477; doi:10.1186/1471-2148-8-60)
Supplement: Additional File 1 — Putative substrate recognition sites (SRSs). [file 1471-2148-8-60-S1.pdf]

Additional file 1

Putative substrate recognition sites (SRSs) 1 to 6.

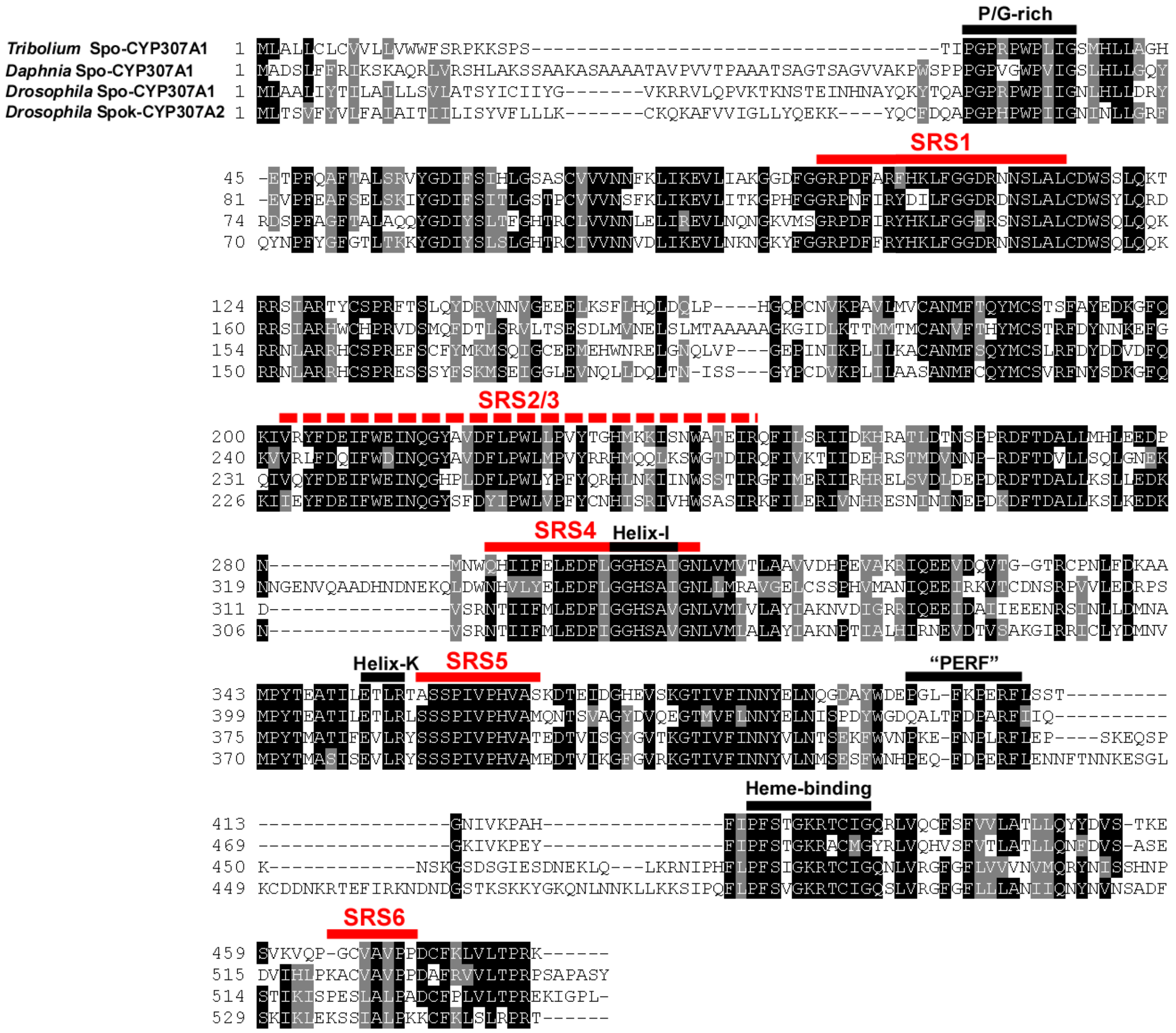

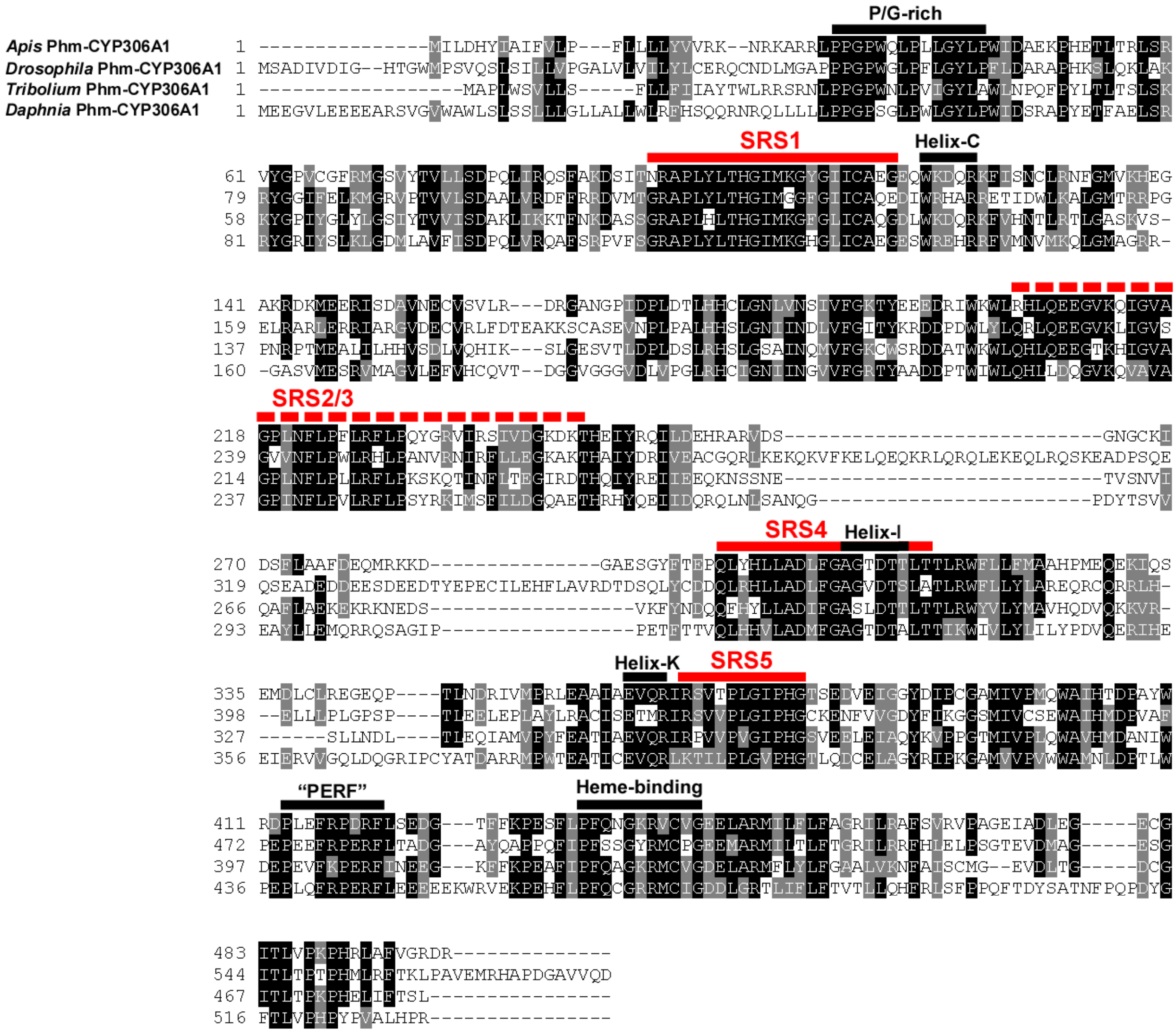

P/G-rich
Apis Phm-CYP306A1
Drosophila Phm-CYP306A1
Tribolium Phm-CYP306A1
Daphnia Phm-CYP306A1
SRS1
Helix-C
SRS2/3
SRS4
Helix-I
Helix-K
SRS5
"PERF"
Heme-binding

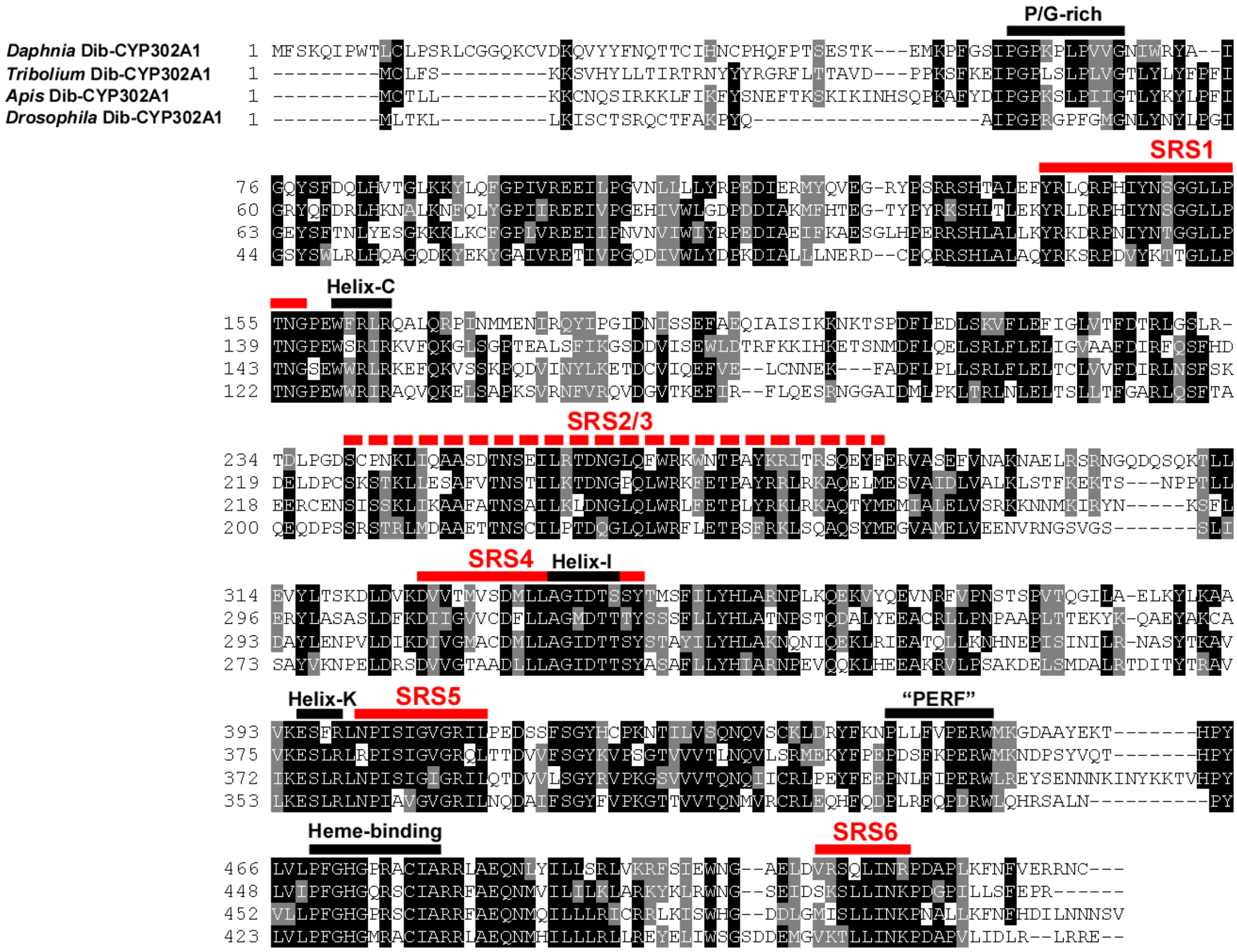
P/G-rich
Daphnia Dib-CYP302A1 1 MFSKQIPWTLCLPSRLCGGQKCVDKQVYYFNQTTCIHNCPHQFPTSESTK---EMKPFGSIPGPKPLPVVGNIWRYA--I
Tribolium Dib-CYP302A1 1 ---------MCLFS---------KKSVHYLLTIRTRNYYRGRFLTTAVD---PPKSFKEIPGPLSLPLVGTLYLYFPFI
Apis Dib-CYP302A1 1 ---------MCTLL---------KKCNQSIRKKLFIKFYSNEFTKSKIKINHSQPKAFYDIPGPKSLPIIGTLYKYLPFI
Drosophila Dib-CYP302A1 1 ---------MLTKL---------LKISCTSRQCTFAKPYQ-----------------AIPGPRGPFGMGNLYNYLPGI
SRS1
76 GQYSFDQLHVTGLKKYLQFGPIVREEILPGVNLLLLYRPEDIERMYQVEG-RYPSRRSHTALEFYRLQRPHIYNSGGLLP
60 GRYQFDRLHKNALKNFQLYGPIIREEIVPGEHIVWLGDPDDIAKMFHTEG-TYPYRKSHLTLEKYRLDRPHIYNSGGLLP
63 GEYSFTNLYESGKKKLKCFGPLVREEIIPNVNVIWIYRPEDIAEIFKAESGLHPERRSHLALLKYRKDRPNIYNTGGLLP
44 GSYSWLRLHQAGQDKYEKYGAIVRETIVPGQDIVWLYDPKDIALLLNERD--CPQRRSHLALAQYRKSRPDVYKTTGLLP
Helix-C
155 TNGPEWFRLRQALQRPINMMENIRQYIPGIDNISSEFAEQIAISIKKNKTSPDFLEDLSKVFLEFIGLVTFDTRLGSLR-
139 TNGPEWSRIRKVFQKGLSGPTEALSFIKGSDDVISEWLDTRFKKIHKETSNMDFLQELSRLFLELIGVAAFDIRFQSFHD
143 TNGSEWWRLRKEFQKVSSKPQDVINYLKETDCVIQEFVE--LCNNEK---FADFLPLLSRLFLELTCLVVFDIRLNSFSK
122 TNGPEWWRIRAQVQKELSAPKSVRNFVRQVDGVTKEFIR--FLQESRNGGAIDMLPKLTRLNLELTSLLTFGARLQSFTA
SRS2/3
234 TDLPGDSCPNKLIQAASDTNSEILRTDNGLQFWRKWNTPAYKRITRSQEYFERVASEFVNAKNAELRSRNGQDQSQKTLL
219 DELDPCSKSTKLLESAFVTNSTILKTDNGPQLWRKFETPAYRRLRKAQELMESVAIDLVALKLSTFKEKTS---NPPTLL
218 EERCENSISSKLIKAAFATNSAILKLDNGLQLWRLFETPLYRKLRKAQTYMEMIALELVSRKKNNMKIRYN-----KSFL
200 QEQDPSSRSTRLMDAAETTNSCILPTDQGLQLWRFLETPSFRKLSQAQSYMEGVAMELVEENVRNGSVGS-------SLI
SRS4
Helix-I
314 EVYLTSKDLDVKDVVTMVSDMLLAGIDTSSYTMSFILLYHLARNPLKQEKVYQEVNRFVPNSTSPVTQGILA-ELKYLKAA
296 ERYLASASLDFKDIIGVVCDFLLAGMDTTTYSSSFLLYHLATNPSTQDALYEEACRLLPNPAAPLTTEKYK-QAEYAKCA
293 DAYLENPVLDIKDIVGMACDMLLAGIDTTSYSTAYILLYHLAKNQNIQEKLRIEATQLLKNHNEPISINILR-NASYTKAV
273 SAYVKNPELDRSDVVGTAADLLLAGIDTTSYAASAFLLYHIARNPEVQQKLHEEAKRVLPSAKDELSMDALRTDITYTRAV
Helix-K
SRS5
"PERF"
393 VKESFRLNPISIGVGRILPEDSSFSGYHCPKNTILVSQNQVSCKLDRYFKNPLLFVPERWMKGDAAYEKT-------HPY
375 VKESLRLRPISIGVGRQLTTDVVFSGYKVPSGTVVVTLNQVLSRMEKYFPEPDSFKPERWMKNDPSYVQT-------HPY
372 IKESLRLNPISIGIGRILQTDVVLSGYRVPKGSVVVTQNQIICRLPEYFEEPNLFIPERWLREYSENNNKINYKKTVHPY
353 LKESLRLNPIAVGVGRILNQDAIFSGYFVPKGTTVVTQNMVRCRLEQHFQDPLRFQPDRWLQHRSALN----------PY
Heme-binding
SRS6
466 LVLPFGHGPRACIARRLAEQNLYILLSRLVKRFSIEWNG--AELDVRSQLINRPDAPLKFNFVERRNC---
448 LVIPFGHGQRSCIARRFAEQNMVILLTLKLARKYKLRWNG--SEIDSKSLLINKPDGPILLSFEPR------
452 VLLPFGHGPRSCIARRFAEQNMQILLLRICRRLKISWHG--DDLGMISLLINKPNALLKFNFHDILNNNSV
423 LVLPFGHGMRACIARRLAEQNMHILLLRLLREYELIWSGSDDEMGVKTLLINKPDAPVLIDLR--LRRE--

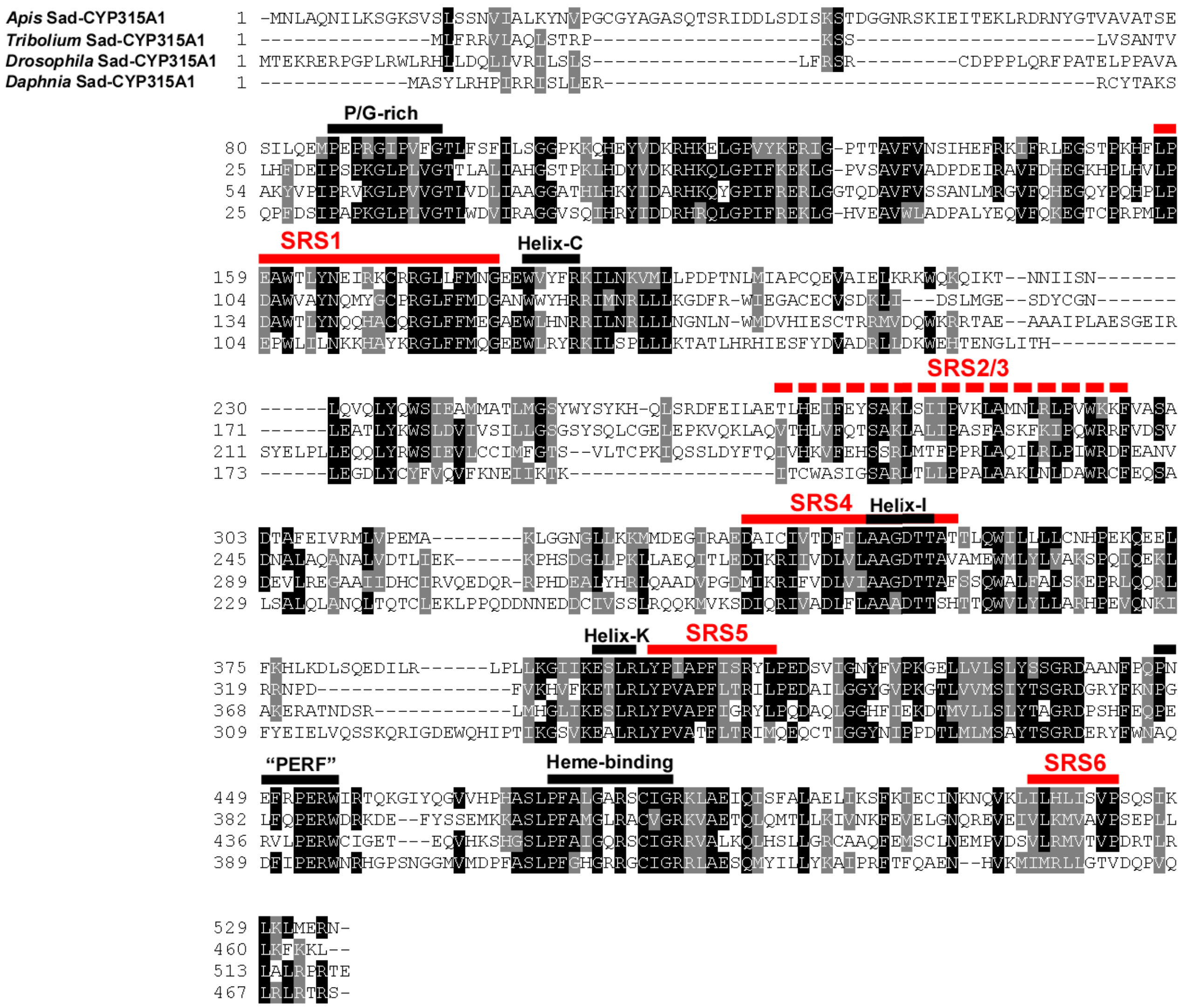
Apis Sad-CYP315A1 1 -MNLAQNILKSGKSVSLSSNVIALKYNVPGCGYAGASQTSRIDDLSDISKSTDGGNRSKIEITEKLRDRNYGTVAVATSE
Tribolium Sad-CYP315A1 1 ----------------MLFRRVIAQLSTRP--------------------KSS---------------------LVSANTV
Drosophila Sad-CYP315A1 1 MTEKRERPGPLRWLRHLLDQLLVRILSLS------------------LFRSR---------CDPPPLQRFPATELPPAVA
Daphnia Sad-CYP315A1 1 -------------MASYLRHPIRRISLLER---RCYTAKS
P/G-rich
80 SILQEMPEPRGIPVFGTLFSFILSGGPKKQHEYVDKRHKELGPVYKERIG-PTTAVFVNSIHEFRKIFRLEGSTPKHFLP
25 LHFDEIPSPKGLPLVGTTLALIAHGSTPKLHDYVDKRHKQLGPIFKEKLG-PVSAVFVADPDEIRAVFDHEGKHPLHVLP
54 AKYVPIPRVKGLPVVGTIVDLIAAGGATHLHKYIDARHKQYGPIFRERLGGTQDAVFVSSANLMRGVFQHEGQYPQHPLP
25 QPFDSIPAPKGLPLVGTLWDVIRAGGVSQIHRYIDDRHRQLGPIFREKLG-HVEAVWLADPALYEQVFQKEGTCPRPMLP
SRS1
Helix-C
159 EAWTLYNEIRKCRRGLLFMNGEEWVYFRKILNKVMLLPDPTNLMIAPCQEVAIELKRKWQKQIKT--NNIISN-------
104 DAWVAYNQMYGCPRGLFFMDGANWWYHRRIMNRLLLKGDFR-WIEGACECVSDKLI---DSLMGE--SDYCGN-------
134 DAWTLYNQQHACQRGLFFMEGAEWLHNRRILNRLLLNGNLN-WMDVHIESCTRRMVDQWKRRTAE--AAAIPLAESGEIR
104 EPWLILNKKHAYKRGLFFMQGEEWLRYRKILSPLLLKTATLHRHIESFYDVADRLIDKWEHTENGLITH-----------
SRS2/3
230 ------LQVQLYQWSIEAMMATLMGSYWYSYKH-QLSRDFEILAETLHEIFEYSAKLSIIPVKLAMNLRLPVWKKFVASA
171 ------LEATLYKWSLDVIVSILLGSGSYSQLCGELEPKVQKLAQVTHLVFQTSAKLALIPASFASKFKIPQWRRFVDSV
211 SYELPLLEQQLYRWSIEVLCCIMFGTS--VLTCPKIQSSLDYFTQIVHKVFEHSSRLMTFPPRLAQILRLPIWRDFEANV
173 ------LEGDLYCYFVQVFKNEIIKTK-----------------ITCWASIGSARLTLLPPALAAKLNLDAWRCFEQSA
SRS4
Helix-I
303 DTAFEIVRMLVPEMA--------KLGGNGLLKKMMDEGIRAEDAICIVTDFILAAGDTTATTLQWILLLLCNHPEKQEEL
245 DNALAQANALVDTLIEK------KPHSDGLLPKLLAEQITLEDIKRIIVDLVLAAGDTTAVAMEWMLYLVAKSPQIQEKL
289 DEVLREGAAIIDHCIRVQEDQR-RPHDEALYHRLQAADVPGDMIKRIFVDLVIAAGDTTAFSSQWALFALSKEPRLQQRL
229 LSALQLANQLTQTCLEKLPPQDDNNEDDCIVSSLRQQKMVKSDIQRIVADLFLAAADTTSHTTQWVLYLLARHPEVQNKI
Helix-K
SRS5
375 FKHLKDLSQEDILR------LPLLKGIIKESLRLYPIAPFISRYLPEDSVIGNYFVPKGELLVLSLYSSGRDAANFPQPN
319 RRNPD-----------------FVKHVFKETLRLYPVAPFLTRILPEDAILGGYGVPKGTLVVMSIYTSGRDGRYFKNPG
368 AKERATNDSR------------LMHGLIKESLRLYPVAPFIGRYLPQDAQLGGHFIPKDTMVLLSLYTAGRDPSHFEQPE
309 FYEIELVQSSKQRIGDEWQHIPTIKGSVKEALRLYPVATFLTRIMQEQCTIGGYNIPPDTLMLMSAYTSGRDERYFWNAQ
"PERF"
Heme-binding
SRS6
449 EFRPERWIRTQKGIYQGVVHPHASLPFALGARSCIGRKLAEIQISFALAELIKSFKIECINKNQVKLILHLISVPSQSIK
382 LFQPERWDRKDE--FYSSEMKKASLPFAMGLRACVGRKVAETQLQMTLLKIVNKFEVELGNQREVEIVLKMVAVPSEPLL
436 RVLPERWCIGET---EQVHKSHGSLPFAIGQRSCIGRRVALKQLHSLLGRCAAQFEMSCLNEMPVDSVLRMVTVPDRTLR
389 DFIPERWNRHGPSNGGMVMDPFASLPFGHGRRGCIGRRLAESQMYILLYKAIPREFTFQAEN--HVKMIMRLLGTVDQPVQ
529 LKLMERN-
460 LKFKKL--
513 LALRPRTE
467 LRLRTRS-

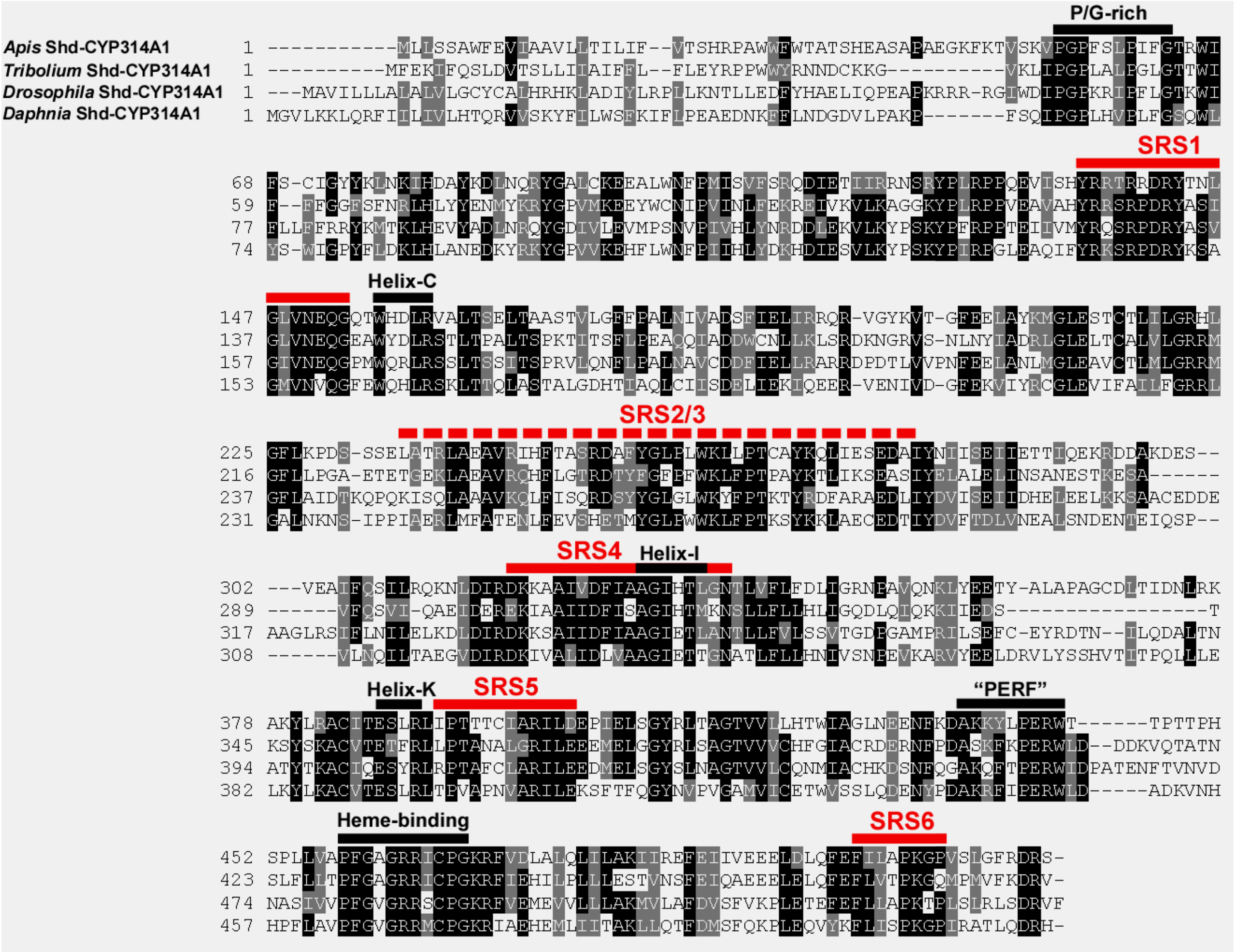

P/G-rich
Apis Shd-CYP314A1
Tribolium Shd-CYP314A1
Drosophila Shd-CYP314A1
Daphnia Shd-CYP314A1
SRS1
Helix-C
SRS2/3
SRS4
Helix-I
Helix-K
SRS5
"PERF"
Heme-binding
SRS6
