# Supplementary material for: Daphnia Halloween genes that encode cytochrome P450s mediating the synthesis of the arthropod molting hormone: Evolutionary implications
Source: BMC Evol Biol. 2008 Feb 25;8:60. doi: 10.1186/1471-2148-8-60 (PMC2276477; doi:10.1186/1471-2148-8-60)
Supplement: Additional File 2 — Deduced protein sequences of Daphnia pulex Spo-CYP307A1, Phm-CYP306A1, Dib-CYP302A1, Sad-CYP315A1 and Shd-CYP314A1. [file 1471-2148-8-60-S2.pdf]

Additional file 2

Deduced protein sequences of *Daphnia pulex* Spo-CYP307A1, Phm-CYP306A1, Dib-CYP302A1, Sad-CYP315A1 and Shd-CYP314A1. Information about the genomic sequences and protein ID obtained at The DOE Joint Genome Institute [http://www.jgi.doe.gov/]. Introns are indicated by (0), phase 0 intron; (1), phase 1 intron; (2), phase 2 intron.

***Daphnia* Spook (Spo: CYP307A1):**
(Dappu1/scaffold_4:1888919-1892238; Protein ID 41863)
MADSLFFRIKSKAQRLVRSHLAKSSAAKASAAAATAVPVVTPAAATSAGTSAGVVAKPWSPPPGPVGWPVIGSLHLLGQY
EVPFEAFSELSKIYGDIFSITLGSTPCVVVNSFKLIKEVLITKGPHFGGRPNFIRYDILFGGDRDN(1)SLALCDWSYLQ
RDRRSIARHWCHPRVDSMQFDTLSRVLTSESDLMVNELSLMTAAAAAGKGIDLKTTMMTMCANVFTHYMCSTRFDYNNKE
FGKVVRLFDQIFWDINQGYAVDFLPWLMPVYRRHMQQLKSWGTDIRQFIVKTIIDEHRSTMDVNNPRDFTDVLLSQLGNE
KNNGENVQAADHNDNEKQLDWNHVLYELEDFLGGHSAIGNLLMRAVGELCSSPHVMANIQEEIRKVTCDNSRPVVLEDRP
SMPYTEATILETLRLSSSPIVPHVAMQNTSVA(1)GYDVQEGTMVFLNNYELNISPDYWGDQALTFDPARFIIQGKIVKP
EYFIPFSTGKRACMGYRLVQHVSFVTLATLLQNFDVSASEDVIHLPKACVAVPPDAFRVVLTPRPSAPASY

***Daphnia* Phantom (Phm: CYP306A1):**
(Dappu1/scaffold_10:1477916-1482392; Protein ID 46010)
MEEGVLEEEEARSVGVWAWLSLSSLLLGLLALLWLRFHSQQRNRQLLLLLPPGPSGLPWLGYLPWIDSRAPYETFAELSR
RYGRIYSLKLGDMLAVFISDPQLVRQAFSRPVFSGRAPLYLTHGIMKGH(1)GLICAEGESWREHRRFVMNVMKQLGMAG
RRGASVMESRVMAGVLEFVH(0)CQVTDGGVGGGVDLVPGLRHCIGNIINGVVFGRTYAADDPTWIWLQHLLDQGVKQVA
VAGPINFLPVLR(2)FLPSYRKIMSFILDGQAETHRHYQEIIDQRQLNLSANQG(1)PDYTSVVEAYLLEMQRRQSAGIP
PETFTTVQLHHVLADMFGAGTDTALTTIKWIVLYLILYPDVQ(0)ERIHEEIERVVGQLDQGRIPCYATDARRMPWTEAT
ICEVQRLKTILPLGVPHGTLQ(0)DCELAGYRIPKGAMVVPVWWAMNLDPTLWPEPLQFRPERFL(0)EEEEEKWRVEKP
EHFLPFQCGRRMCIGDDLGRTLIFLFTVTLLQHFRLSFPPQFTDYSATNFPQPDYGFTLVPHPYPVALHPR

***Daphnia* Disembodied (Dib: CYP302A1):**
(Dappu1/scaffold_811:2049-4377; Protein ID 204789)
MFSKQIPWTLCLPSRLCGGQKCVDKQVYYFNQTTCIHNCPHQFPTSESTKEMKPFGSIPGPKPLPVVGNIWRYAI(1)GQ
YSFDQLHVTGLKKYLQFGPIVREEILPGVNLLLLYRPEDIERMYQVEGRYPSRRSHTALEFYRLQRPHIYNSGGLLPT(2
)NGPEWFRLRQALQRPINMMENIRQYIPGIDNISSEFAEQIAISIKKNKTSPDFLEDLSKVFLE(1)FIGLVTFDTRLGS
LRTDLPGDSCPNKLIQAASDTNSEILRTDNGLQFWRKWNTPAYKRITRSQEYFER(2)VASEFVNAKNAELRSRNGQDQS
QKTLLEVYLTSKDLDVKDVVTMVSDMLLAGIDT(0)SSYTMSFILYHLARNPLKQEKVYQEVNRFVPNSTSPVTQGILAE
LKYLKAAVKESFRLNPISIGVGRILPEDSSFSGYHCPKN(0)TILVSQNQVSCKLDRYFKNPLLFVPERWMKGDAAYEKT
HPYLVLPFGHGPRACIARRLAEQNLYILLSR(0)LVKRFSIEWNGAELDVRSQLINRPDAPLKFNFVERRNC

***Daphnia* Shadow (Sad: CYP315A1):**
(Dappu1/scaffold_93:309807-311873; Protein ID 60760)
MASYLRHPIRRISLLERRCYTAKSQPFDSIPAPKGLPL(0)VGTLWDVIRAGGVSQIHRYIDDRHRQLGPIFREKLGHVE
AVWLADPALYEQVFQKEGTCPRPMLPEPWLILNKKHAYKRGLFFM(2)QGEEWLRYRKILSPLLLKTATLHRHIESFYDV
ADRLLDKWEHTENGLITHLEGDLYCYFVQ(0)VHILVLVNWLNQEFYMNRNFRIIKTKITCWASIGSARLTLLPPALAAK
LNLDAWRCFEQSALSALQLANQLTQTCLEKLPPQDDNNEDDCIVSSLRQQKMVKSDIQRIVADLFLAAADT(0)TSHTTQ
WVLYLLARHPEVQNKIFYEIELVQSSKQRIGDEWQHIPTIKGSVKEALRLYPVATFLTRIMQEQCTIGGYNIPPD(0)TL
MLMSAYTSGRDERYFWNAQDFIPERWNRHGPSNGGMVMDPFASLPFGHGRRGCIGRRLAESQMYILLYK(0)AIPRFTFQ
AENHVKMIMRLLGTVDQPVQLRLRTRS

***Daphnia* Shade (Shd: CYP314A1):**
(Dappu1/scaffold_43:996318-999232; Protein ID 198743)
MGVLKKLQRFIILIVLHTQRVVSKYFILWSFKIFLPEAEDNKFFLND(1)GDVLPAKPFSQIPGPLHVPLFGSQWLYSWI
GPYFLDKLHLANE(1)DKYRKYGPVVKEHFLWNFPIIHLYDKHDIESVLKYPSKYPIRPGLEAQIFYRKSRPDRYK(1)S
AGMVNV(2)QGFEWQHLRSKLTTQLASTALGDHTIAQLCIISDELIEKIQEERVENIVDGFEKVIYRCGLE(1)VIFAIL
FGRRLGALNKNSIPPIAERLMFATENLFEVSHETMYGLPWWKLFPTKSYKKLAECEDTIYD(2)VFTDLVNEALSNDENT
EIQSPVLNQILTAEGVDIRDKIVALIDLVAAGIET(0)TGNATLFLLHNIVSNPEVKARVYEELDRVLYSSHVTITPQLL
LELKYLKACVTESLR(2)LTPVAPNVARILEKSFTFQGYNVPVG(0)AMVICETWVSSLQDENYPDAKRFIPERWLDADK
VNHHPFLAVPFGVGRRMCPGKRIAEHEMLIITAK(0)LLQTFDMSFQKPLEQVYKFLISPKGPIRATLQDRH
